# Neurodevelopmental LincRNA Microsyteny Conservation and Mammalian Brain Size Evolution

**DOI:** 10.1371/journal.pone.0131818

**Published:** 2015-07-02

**Authors:** Eric Lewitus, Wieland B. Huttner

**Affiliations:** Max Planck Institute of Molecular Cell Biology and Genetics, Pfotenhauerstr. 108, 01307 Dresden, Germany; National Center for Biotechnology Information, UNITED STATES

## Abstract

The mammalian neocortex has undergone repeated selection for increases and decreases in size and complexity, often over relatively short evolutionary time. But because probing developmental mechanisms across many species is experimentally unfeasible, it is unknown whether convergent morphologies in distantly related species are regulated by conserved developmental programs. In this work, we have taken advantage of the abundance of available mammalian genomes to find evidence of selection on genomic regions putatively regulating neurogenesis in large- *versus* small-brained species. Using published fetal human RNA-seq data, we show that the gene-neighborhood (i.e., microsynteny) of long intergenic non-coding RNAs (lincRNAs) implicated in cortical development is differentially conserved in large-brained species, lending support to the hypothesis that lincRNAs regulating neurogenesis are selectively lost in small-brained species. We provide evidence that this is not a phenomenon attributable to lincRNA expressed in all tissue types and is therefore likely to represent an adaptive function in the evolution of neurogenesis. A strong correlation between transcription factor-adjacency and lincRNA sequence conservation reinforces this conclusion.

## Introduction

The mammalian neocortex is remarkably diverse. While it shows some general uniformity across species (e.g., a six-layered structure and division into functional areas), it is as varied as the adaptive behaviors it governs [1, 2]. Development of the neocortex, however, like most aspects of development [3], retains a much stricter pattern across species, involving a conserved arsenal of progenitor-types. Indeed, these major progenitor-types—apical radial glia (aRG) and basal radial glia (bRG), as well as apical and basal intermediate progenitors (IPs)—are putatively present in all mammals [4, 5, 6, 7]. But in those mammals with larger, convoluted neocortices (i.e., gyrencephalic species), a heterogeneity of bRGs is observed [8], and an increased proliferative potential in basally dividing progenitors is important for cortical size and folding [4, 6, 9, 10, 11, 12, 13]. In addition, recent work has shown that, both neuroanatomically and neurodevelopmentally, mammals may be segregated into two principal groups, delimited by a threshold gyrencephaly index (GI) value of 1.5 (corresponding to approximately one billion cortical neurons) [12]. Thus, we may define species as being high-GI or low-GI. But despite these categorical differences, species differences in cortical development at the genomic level have been given surprisingly little attention [14, 15, 16, 17, 18, 19] and virtually no attention across all mammalian orders [20, 21]. It is largely unknown, therefore, how neurogenesis has evolved in mammals to generate so many radical increases and decreases in neocortical size—or even whether any general principles of building bigger brains can be found across disparate clades.

Here, in order to assess the degree to which neocortical convergence, both in the generation of certain neural progenitor-types and the presentation of cortical growth and folding above a threshold value, is corroborated by convergence at the genomic level, we probed published RNA-seq data collected from human fetal neocortical germinal zones during neurogenesis [22]. Because lincRNAs show accelerated evolution in humans [23], have high levels of tissue- and age-specificity [24], and are potential developmental regulators [25, 26, 27], we limited our probes to lincRNAs. We did this, furthermore, because protein and transcript abundance are poorly correlated [28], at least among closely related species, thus making it difficult to interpret the significance of coding-gene expression for explaining interspecific phenotypic differences. We show that the ancestral gene-neighborhoods of lincRNAs implicated in cortical development (neurodevelopmental lincRNAs), in contrast to lincRNAs predominantly expressed in other tissues, are selectively lost in small-brained species. We argue that this supports not only a functional role for lincRNAs in mammalian neurogenesis [29], but an adaptive role for lincRNAs in neocortical evolution.

## Materials and Methods

**Fig 1:**
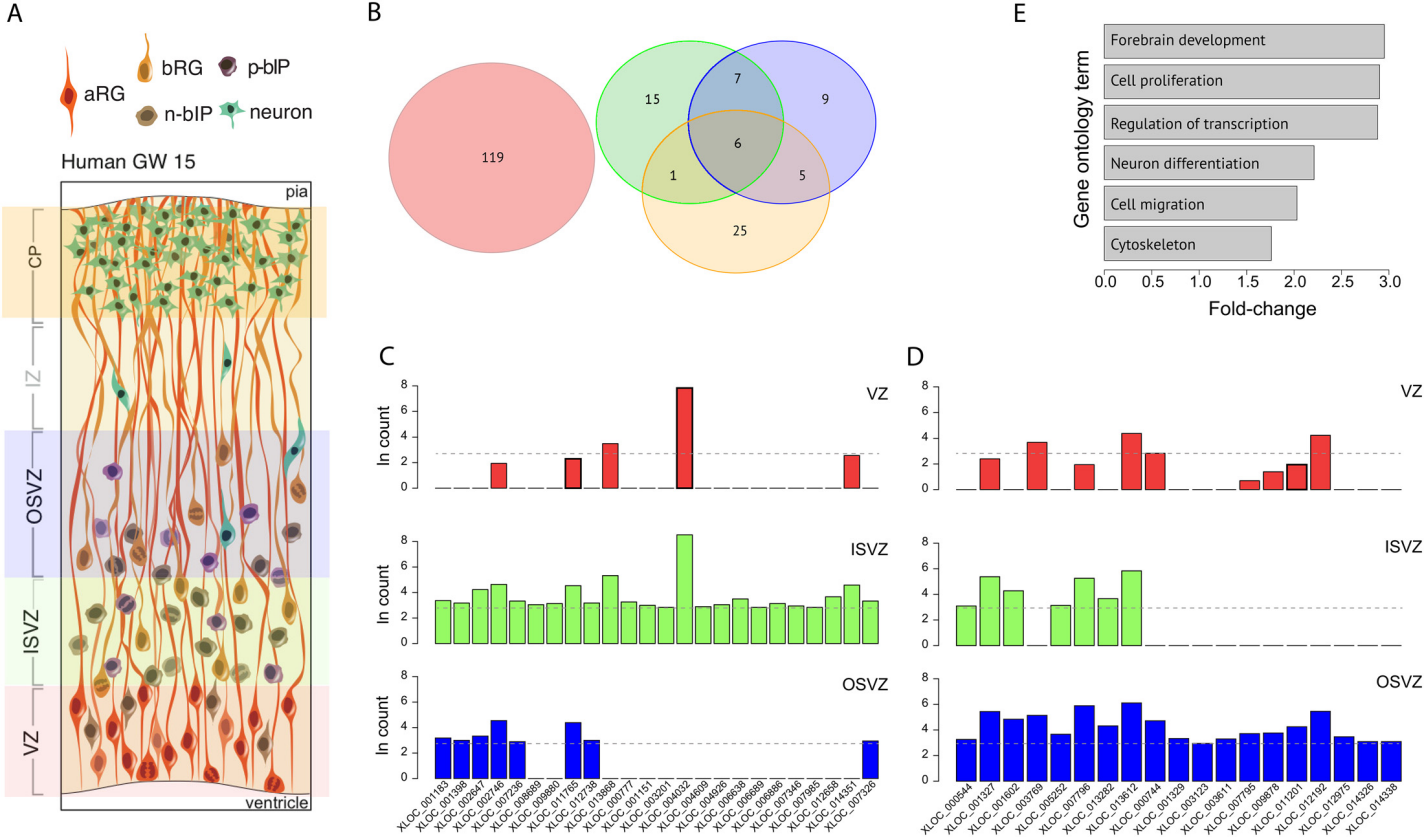
Germinal zone-specific transcript levels of lincRNAs in GW13–16 human neocortex as determined by RNA-seq. (A) Schematic of human germinal zones dissected for RNA-seq [22], depicting aRG, bRG, neurogenic basal intermediate progenitors (n-bIPs), proliferative basal intermediate progenitors (p-bIPs), and neurons. Adapted from [9]. (B) Number of differentially expressed lincRNAs in each germinal zone (VZ, red; ISVZ, green; OSVZ, blue; CP, orange). Analyses were run for each layer against the VZ. (C,D) LincRNAs differentially overexpressed in the (C) ISVZ and (D) OSVZ. The dashed grey line shows the mean transcript level for lincRNAs overexpressed in the VZ. (E) LincRNAs expressed during human neurogenesis tend to have gene-adjacent neighbors involved in neocortical development. Shown are fold-enrichments of Gene Ontology (GO) terms for adjacent protein-coding gene neighbors of the 142 lincRNAs expressed during human neurogenesis (see S1 Table). GO terms are listed if they are over-represented in the protein-coding gene set (FDR < 0.05). Fold differences for enriched GO terms were analyzed using DAVID (http://david.abcc.ncifcrf.gov/summary.jsp) with the entire set of genes expressed in the fetal brain [22] as a base.

We used previously published RNA-seq data (Series GSE38805) collected from the ventricular zone (VZ), inner subventricular zone (ISVZ), outer subventricular zone (OSVZ), and cortical plate (CP) of human fetal neocortex at gestation week (GW)13–16 [22] and employed the lincRNA discovery pipeline outlined by [30] to identify 187 lincRNA differentially expressed during human neocortical neurogenesis (Fig 1A–1D, S1 Table). We aligned those lincRNAs found in our dataset with those deposited at the Human Body Map lincRNAs, so that all XLOC IDs are preserved from [30]. Of these, 161 were differentially upregulated in a germinal zone, including 43 overexpressed in the ISVZ and/or OSVZ compared to the VZ (Fig 1B–1D). Fifty-seven of the 161 lincRNAs are also relatively highly expressed in the adult brain [30]; and, by comparing names and/or start-sites for our lincRNAs with those identified in recent work on lincRNA expression in tetrapods, we found 52 to be putatively conserved across primates or eutheria according to sequence similarity [25]. All differential expression analyses were run with the R package DESeq [31].

Previous work has shown that sequence conservation is a poor predictor of functional conservation in non-coding RNA [32] and that long non-coding RNAs are often functionally—or at least transcriptionally—linked to adjacently located protein-coding genes [33, 34]. Therefore, we analyzed lincRNA conservation as a function of gene-neighborhood (i.e., microsynteny). For each lincRNA, we defined its gene-neighborhood as the immediately flanking protein-coding genes and discarded any lincRNAs which did not have at least one flanking gene expressed during neurogenesis. Orthologous flanking genes were identified using Orthomam v8.0 [35]; if they could not be found there, then 1-to-1 orthologs were identified in Ensembl.

The greatest distance between a lincRNA and its nearest neighbor was 22.3 Mb (XLOC_000380), although the median distance was 46 kb. The final list included 142 lincRNAs, whose gene-neighborhoods were collectively enriched for Gene Ontology terms related to forebrain development and cell proliferation (S1 Table; Fig 1E).

**Fig 2:**
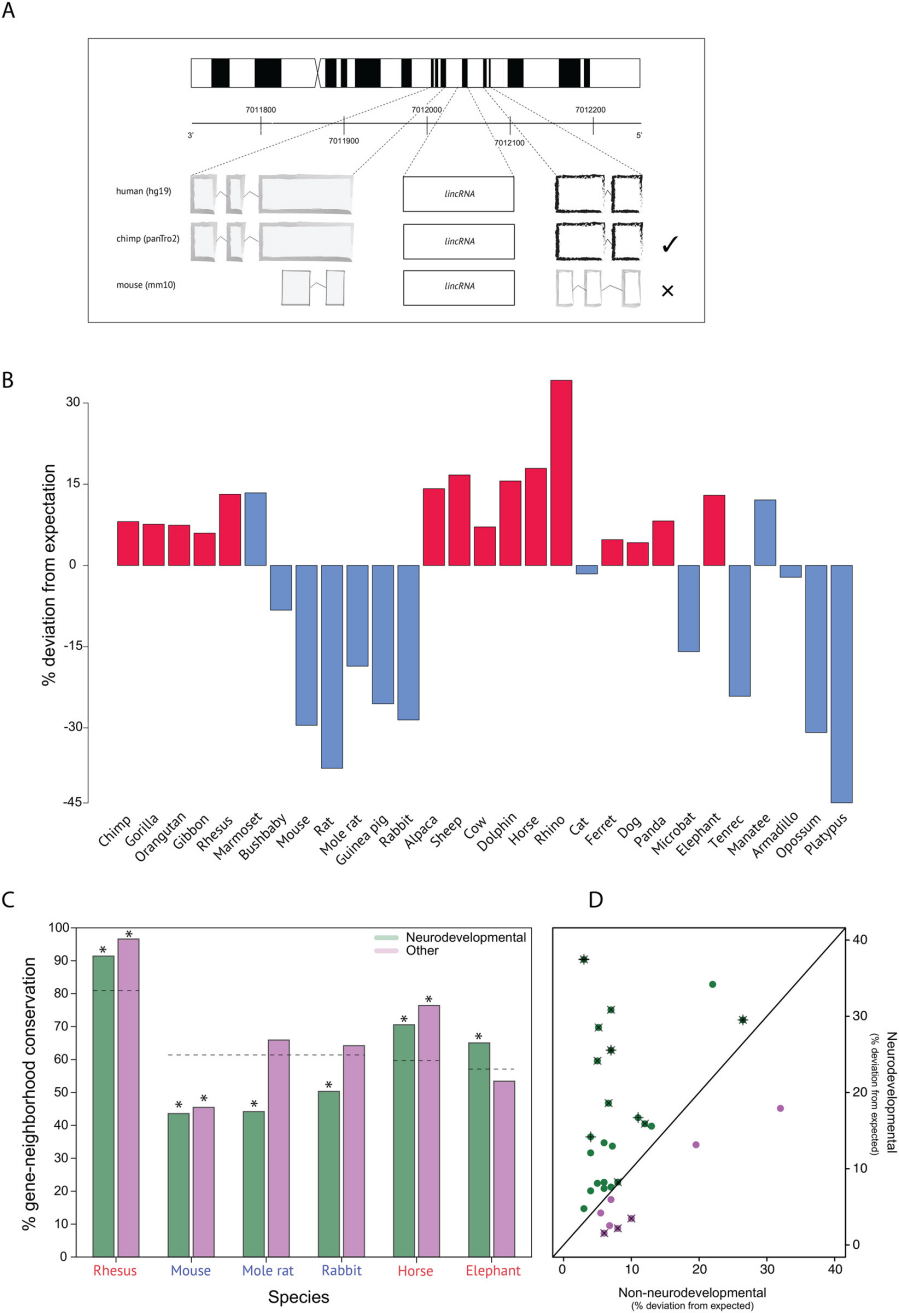
LincRNA gene-neighborhood conservation in neurodevelopmental and non-neurodevelopmental tissue. (A) Schematic of the protocol used for determining lincRNA gene-neighborhood conservation across species. In the example given, only chimp is scored as conserved. Chromosomal synteny is not a condition for gene-neighborhood conservation. (B) Gene-neighborhood conservation of 142 lincRNAs expressed during human neurogenesis across 29 mammalian species (S2 Table). Gene-neighborhood conservation is shown to be above null phylogenetic expectations in high-GI species (red) and below expectations for low-GI species (blue). The two exceptions are the marmoset, a low-GI primate, and the manatee, a large-brained lissencephalic Afrotherian; both of these show lincRNA gene-neighborhood conservation considerably above null expectations The chicken is not shown. (C) Gene-neighborhood conservation for neurodevelopmental lincRNAs (green) and lincRNAs showing maximum levels of expression in non-brain human tissue (lilac) for three high-GI (red) and three low-GI (blue) species. Conservation in the naked mole rat, rabbit, and elephant are significantly different for neurodevelopmental compared to non-brain (Other) lincRNAs, while similar levels of conservation are observed for both classes of lincRNAs in macaque, mouse, and horse. Predicted conservation values for each species (dashed lines) were calculated from null evolutionary models based on divergence times with human. Asterisks denote significantly different values from predicted (P < 0.05). (D) Plot of neurodevelopmental *versus* non-neurodevelopmental (absolute) conservation deviation scores based on a phylogenetic expectation model (see Materials and Methods) for 29 mammalian species. From the regression slope delineating no deviation from expected scores, it is clear that neurodevelopmental lincRNAs deviate more frequently and more sizeably from conservation expectations than non-neurodevelopmental lincRNAs. Crosses and Xs indicate negative deviations for neurodevelopmental and non-neurodevelopmental lincRNAs, respectively. (B) is adapted from [20].

We assessed lincRNA gene-neighborhood conservation for the 142 lincRNAs in humans and 30 other species (see S2 Table) by BLASTing the lincRNA sequence retrieved from the human RNA-seq data and visually inspecting (using the UCSC Genome Browser) the gene-neighborhood for each species (Fig 2A). Regions with the maximum BLASTn alignment score, E-value < 1 × 10^−4^, and query cover > 20% were selected. When BLASTn was unsuccessful in returning any sequences matching these criteria, discontiguous megablast was used. To increase the likelihood of finding an orthologous region in non-human species, we used both the entire lincRNA sequence, as well as, when available, only the region of the lincRNA showing signs of transcriptional activity in human as evidenced by ENCODE data on chromatin-level information [36]. Both sequences consistently identified the same region in non-human species. Due to considerable scaffolding at the time of the analysis, we were only able to assess a portion of the lincRNAs (66) for the dolphin.

Conservation scores for each lincRNA of a given species were tallied as either conserved (1; at least one conserved neighbor) or not conserved (0) (S2 Table). Expectation scores were then calculated under an Ornstein-Uhlenbeck model with a single optimum based on phylogenetic generaliized least squares [37] using three 102-species pruned mammalian supertrees [12, 38] and R package *geiger* [39]. The percentage deviations of actual from expected scores for each species are presented in Fig 2B. GI values were collected from [12].

178 lincRNAs showing their highest levels of expression in non-brain human tissue were collected from [30] and analyzed as above for 30 species (Fig 2C and 2D). Rates of molecular evolution for different species were collected from [40] and used to assess the degree to which different molecular rates across species might confound adaptive explanations for lincRNA gene-neighborhood evolution (S1 Fig).

**Fig 3:**
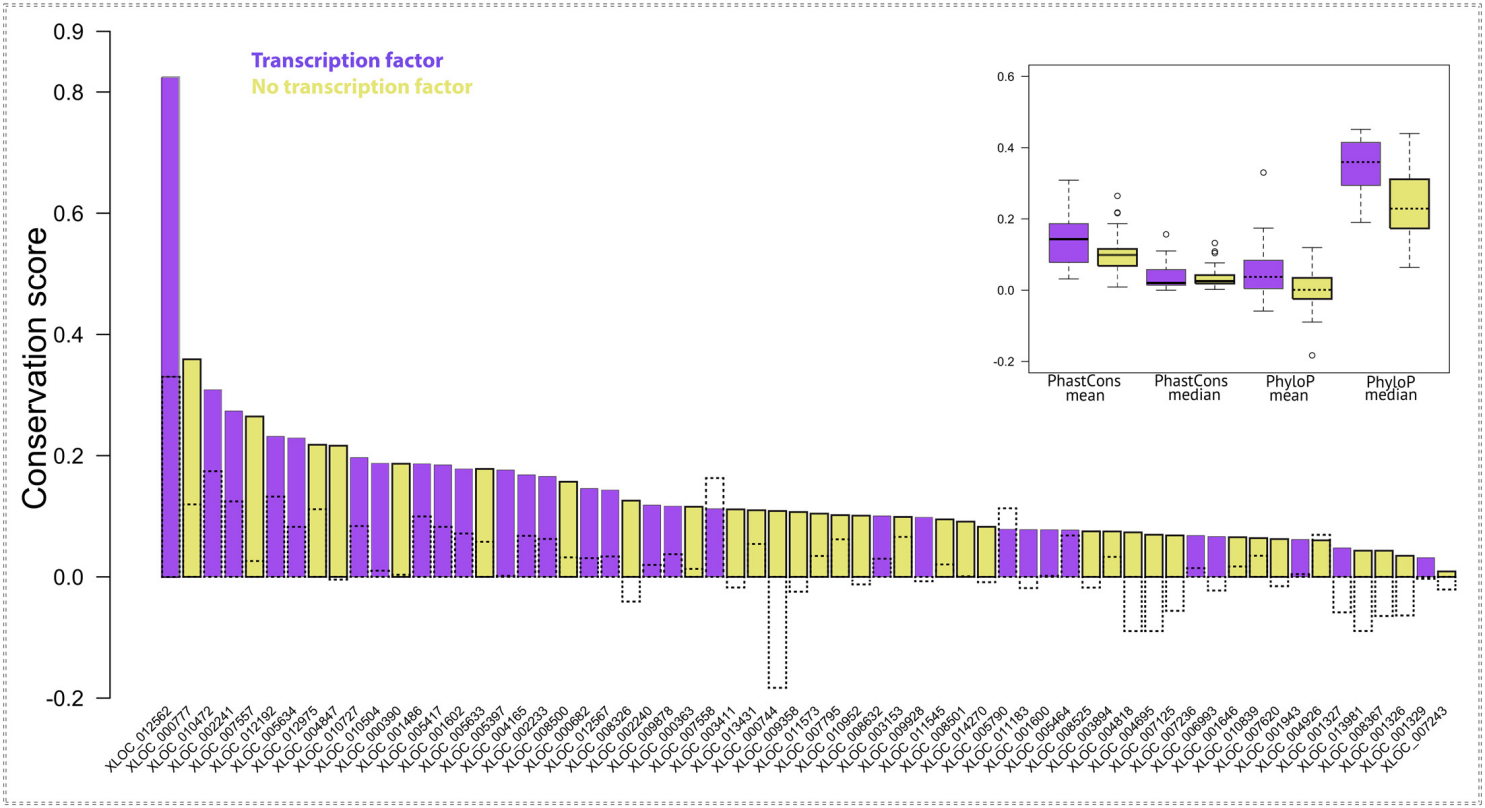
Sequence conservation among primates for lincRNAs expressed during human neurogenesis tends to be higher for lincRNAs flanked by at least one transcription factor. PhastCons scores are shown for 62 lincRNAs, whose gene-neighborhoods are not conserved in mouse (S2 Table). PhyloP scores for the same lincRNAs are shown as dotted bars. (Inset) Boxplot of the mean and median PhyloP and PhastCons scores for primates for the 62 lincRNAs. Mean conservation scores between lincRNAs with (purple) and without (brown) at least one adjacent transcription factor were significantly different for both PhastCons (T = -2.371, P = 0.023) and PhyloP (T = -3.513, P = 0.001), but median scores were significantly different only for PhyloP (T = -5.211, P < 0.001).

Because sequence conservation was likely to be more constrained in closely related species, we computed PhyloP and PhastCons sequence conservation scores for primates for lincRNAs showing gene-neighborhood conservation in primates but not in mouse (S3 Table) [41]. These were used to assess the sequence-level conservation in primates for lincRNAs with and without transcription factor-adjacency (Fig 3).

## Results

We identified 187 lincRNAs differentially expressed in a germinal zone or the cortical plate (CP) of the embryonic human neocortex (Fig 1A–1D, S1 Table). Of these, we shortlisted 142, which had at least one adjacent protein-coding gene also expressed during human neurogenesis (Fig 1E). We then determined whether the genes immediately flanking the lincRNA in the human genome (defined as the lincRNA gene-neighborhood; Fig 2A) were similarly flanking the orthologous genomic sequence in 30 other species (29 mammals plus chicken). We found, firstly, that lincRNA gene-neighborhood conservation could not be explained by phylogenetic relatedness (Fig 2A–2C). By calculating the number of lincRNAs expected to be conserved in each species based on phylogenetic relatedness to human, we could determine which species fell below and which above null expectations. We found that all low-GI species fell below and all high-GI species above phylogenetic expectations (Fig 2B), with two exceptions: the marmoset, a near-lissencephalic primate that is hypothesized to have recently evolved from a gyrencephalic ancestor and therefore may still be in the process of purging unneeded neurodevelopmental lincRNAs [42]; and the manatee, a large-brained (382g), albeit lissencephalic species [43]. Importantly, we found no similar pattern of conservation with non-neurodevelopmental lincRNAs [30] (Fig 2C and 2D); no significant correlation between rate of molecular evolution and neurodevelopmental lincRNA gene-neighborhood conservation (P > 0.1); and GI to be a stronger predictor (*R* ^2^ = 0.68, P < 0.001) than body weight (Z = 1.967, P = 0.025, one-tailed) or longevity (Z = 2.105, P = 0.018, one-tailed) of gene-neighborhood conservation. We therefore provide evidence for a possible genomic correlate of the GI threshold [12] in the disproportionate conservation of neurodevelopmental lincRNAs in high- *versus* low-GI species.

Secondly, we found that, in primates, sequence conservation was highest for gene-neighborhoods containing at least one transcription factor (Fig 3). Mean conservation scores between lincRNAs with and without at least one adjacent transcription factor were significantly different for both PhastCons and PhyloP measures of sequence conservation. These results are in line with increasing evidence for the role of lincRNAs in neurodevelopment as important transcriptional regulators (e.g., [44]). Among large-brained primates, interspecific discrepancies in the timing of neurogenesis are largely a matter of scale, rather than a rearrangement of transcriptional events [45]. It is possible that, at this close phylogenetic range, there has been a strong selection pressure to conserve lincRNAs, even at the primary sequence level, which act as regulatory elements of transcription factors during neurogenesis.

## Discussion

The adaptation of proliferative basal progenitors may be tantamount to a relaxation of constraints along lineages leading to larger-brained species [46]. However, in light of the evidence presented here, we think that convergent gain-of-function along lineages leading to large-brained species is unrealistic. Rather, our analysis of lincRNA gene-neighborhood conservation suggests that the selective loss of genomic elements regulating neurogenesis may be responsible for the evolution of smaller brains in mammals. This means that the genomic developmental toolbox necessary for adapting proliferative basal progenitors, leading to increases in neocortical size and folding, is ancestral to eutherian mammals. Our definition of conservation in terms of microsyteny, rather than primary sequence similarity, allows for such conservation of lincRNAs over extended evolutionary time periods, despite the well-known phenomenon of rapid sequence changes in lincRNAs [47, 48]. Of course, it could be argued that the loss of lincRNAs in low-GI species, which are typically small-bodied, may simply be caused by a higher rate of meiotic recombination in low-GI species, resulting in more frequent meiotic errors and thereupon loss of lincRNAs. However, several lines of evidence presented here make this unlikely to be the case: GI is a better predictor (R^2^ = 0.68, P < 0.001) than lifespan (*R* ^2^ = 0.44, P < 0.001) or body weight (*R* ^2^ = 0.39, P < 0.001) of lincRNA gene-neighborhood conservation [20]; in non-neurodevelopmental lincRNAs, gene-neighborhood conservation can generally be explained by phylogenetic relatedness (Fig 2C and 2D); and rates of molecular evolution are not typically faster in low-GI species compared to high-GI species (S1 Fig). Nonetheless, we cannot entirely rule out faster microsynteny evolution in smaller-brained species as a contributing factor—and, indeed, we would expect it to make some contribution—to neurodevelopmental lincRNA conservation in large-brained species. It is also worth noting that a considerable fraction of lincRNAs overlap enhancer regions [30], which allows for the possibility that enhancer-associated RNAs, rather than lincRNAs, are driving the microsynteny conservation we observe. However, because enhancer-associated RNAs are not enriched (Z = 0.213, P = 0.584) in the neurodevelopmental over the non-neurodevelopmental set of lincRNAs (S2 Table), even though both sets are more enriched than average (Z > 2, P < 0.05), we are confident that the observed effect is driven primarily by selection on lincRNA microsyteny. Finally, while we analysed all mammalian species for which genomic data were available, our sample constitutes a minor fraction (< 1%) of all mammalian species, and so we cannot conclusively say that the confidence intervals for GI, lifespan, and body weight as predictors of lincRNA conservation would hold if the other 99% of mammals were analysed.

We think that, because broadly non-functional heritable sequence mutations are more frequent in lincRNAs compared to protein-coding genes, sequence similarity may not be a reliable measure for functional conservation in lincRNAs between distantly related species. Rather, the microsynteny of a lincRNA, particularly if it is cis-acting, may be a better indicator across species of its functional conservation [32].

We therefore hypothesize, given the results presented here, that the selective loss or retention of neurodevelopmental lincRNAs is relevant for neocortical development and evolution. We think this is a first step in uncovering how convergent evolution of a complex structure may have been achieved at the genomic level. Evidence for lincRNA transcriptional activity in other mammalian species will be crucial for taking this hypothesis forward. Ultimately, how such genomic elements function towards neocortical growth and folding will require investigations into the molecular mechanisms of the differentially conserved lincRNAs identified here.

## Supporting Information

S1 Fig lincRNA conservation as a function of rate of molecular evolution. lincRNA conservation scores plotted against paired differences between species-level and mammalian-average molecular rates, as reported by [40]. Separate regression analyses for high-GI species (R2 = -0.06, P = 0.52) and low-GI species (R2 = 0.03, P = 0.30) are also not significant.
(EPS)

S1 Table Differential expression levels between germinal zones of lincRNAs expressed during human neurogenesis. (XLSX)

S2 Table Gene-neighborhood conservation of lincRNAs expressed during human neurogenesis. (XLSX)

S3 Table PhastCons and PhyloP conservation scores for lincRNAs expressed during human neurogenesis (not conserved in mouse). (XLS)
